# Nomogram for customized recurrence prediction in primary non-muscle-invasive bladder cancer based on routine blood and urine parameters

**DOI:** 10.1186/s12894-024-01437-4

**Published:** 2024-03-25

**Authors:** Yi He, Chenxi Pan, Yue Zhang, Meihong Lv, Bo Yang

**Affiliations:** 1 Department of Urology, the Second Affiliated Hospital of Dalian Medical University, Dalian, Liaoning China; 2 Department of Anesthesiology, the First Affiliated Hospital of Dalian Medical University, Dalian, Liaoning China; 3 The First Affiliated Hospital of Dalian Medical University, 222, Zhongshan Road, Xigang District, Dalian, 116011 China; 4 The Second Affiliated Hospital of Dalian Medical University, 467, Zhongshan Road, Shahekou District, Dalian, 116044 China

**Keywords:** Nomogram, Recurrence, Primary non-muscle-invasive bladder cancer

## Abstract

**Purpose:**

A prevalent condition with a high probability of recurrence, non-muscle invasive bladder cancer (NMIBC) necessitates lifetime surveillance. In patients with pathologically confirmed NMIBC, our goal was to create a unique nomogram to predict recurrence after transurethral resection of bladder tumor (TURBT).

**Methods:**

Our institution’s 91 NMIBC patients with complete follow-up data between January 2017 and February 2021 were included in the retrospective analysis. The nomogram predicting the 0.5, 1, 2 and 3-year likelihood of recurrence was created using multivariate Cox proportional hazard models to find the significant determinants of recurrence. Using the concordance index (C-index), calibration curves, receiver operating characteristic (ROC) curves, and decision curve analyses (DCA), we internally validated the nomogram.

**Results:**

The significant factors related to NMIBC recurrence were age, blood platelet count, especially for the urine leukocyte count and mucus filament. The constructed nomogram performed well in the customized prediction of NMIBC recurrence at 6th, 12th, 24th and 36th month, of which the C-index was 0.724. The calibration curve and the ROC curve both validated the prediction accuracy. On DCA, the nomogram presented good net benefit gains across a wide range of threshold probabilities. Furthermore, the Nomogram-related risk score was used to divide the patient population into two groups with significant recurrence disparities.

**Conclusion:**

For the prediction of NMIBC recurrence, our unique nomogram demonstrated a respectable degree of discriminative capacity, sufficient calibration, and considerable net benefit gain. There will be a need for additional internal and external validation.

## Introduction

With an estimated 500,000 new cases and 200 000 deaths worldwide, bladder cancer (BC) is the ninth most prevalent malignancy globally. In the US alone, there are more than 80,000 new cases and 17,000 fatalities per year [1]. BC can be roughly categorized into invasive muscle bladder cancers (MIBC) and non-invasive muscle bladder cancers (NMIBC). NMIBC makes up over 70% of BC on average. About 70% of the NMIBC exhibit Ta lesions, 20% T1 lesions, and 10% carcinoma in situ (CIS, or Tis lesions) [2]. Transurethral resection of bladder tumor (TURBT) is the main method used to diagnose and treat NMIBC [3].

As a result of the heterogeneous nature of NMIBC and its high likelihood of progression and recurrence, practically all NMIBC patients must undergo frequent surveillance after TURBT [4]. Therefore, for the prognosis and economical treatment of the disease, early detection of recurrence is essential. Researchers have created a number of risk prediction methods to help forecast the probability of recurrence following TURBT for NMIBC [5–8]. Diagnostic biomarkers for possible malignancies have been discovered for both blood and urine [9–12], but none of them have been suggested for the follow-up of NMIBC recurrence in clinical practice guidelines.

With continually developing technology, we discovered that standard blood and urine measurements are frequently disregarded when it comes to the prognosis of cancer. Therefore, the purpose of this study was to determine whether blood and urine parameters were related to the prognosis of NMIBC and to develop a unique nomogram for quickly estimating the likelihood of recurrence following TURBT.

## Material and method

### Patients

The medical records of patients who underwent TURBT during 2017 and 2018 were accessed, and their follow-up information from 2017 to 2023 was collected. The inclusion criteria were as follows. (1) Initially pathologically confirmed as NMIBC. (2) Received standard intravesical therapy, including adjuvant BCG and/or other kinds of chemotherapy. (3) With complete clinical and follow-up data. The exclusion criteria were as follows. (1) Locally advanced (T2 or higher), or metastatic bladder cancers. (2) benign lesions (such as papillomas and cystitis). (3) Upper urinary tract urothelial carcinoma (UTUC). (4) Comorbidity of other neoplastic diseases.The protocol was accepted by the ethical committee of the Second Hospital of Dalian Medical University (SAHDMU) with the Declaration of Helsinki (approval number: 2,023,177), all patients submitted written informed consent prior to participation. Cystoscopic examinations or urine cytological examinations were performed every 3 months after surgery for recurrence surveillance. An additional telephone follow-up was conducted for patients who performed examinations in local medical institutions. As a result, 91 patients were included in this retrospective investigation, and all patients were followed up to their first recurrence.

### Assessed clinico-pathological variables

We extracted the following clinico-pathological variables: age at the time of surgery, gender, tumor stage, tumor size (< 3 cm vs. ≥ 3 cm), tumor grade (low vs. high), number of tumors (single vs. multiple), non-pedicled (no vs. yes), blood platelet count and lymphocyte count, serum albumin and haemoglobin values, urinary leucocyte counts, nitrite, protein, bacterium and mucus filament. HALP score was calculated as hemoglobin (g/L) × albumin (g/L) levels × lymphocyte count (/L) / platelet count (/L). Based on the results of the preoperative computed tomography or cystoscopic examination, the tumor size, non-pedicled, and number of each case were evaluated. In compliance with a structured reporting routine, all TURBT specimens had been handled according to our institutional pathology practices and evaluated by knowledgeable genitourinary pathologists. The American Joint Committee on Cancer staging system from 2010 was used to assess the tumor’s stage. Based on World Health Organization systems from 2004, the tumor grade was determined.

### Establish a nomogram for predicting the NMIBC recurrence

To incorporate data on survival time, survival status, and clinico-pathological features, the R package “survival” was employed. The predictive importance of these factors was determined using univariate and multivariate Cox regression analysis. A nomogram predicting disease recurrence rates at 6, 12, 24, and 36 months was produced according to multivariate Cox proportional hazards analysis using the “RMS” software. The nomogram presents graphical data for these factors, and from the points linked with each risk factor, the prognosis risk of an individual patient may be computed. During nomogram validation, the total points of each patient in the validation cohort were determined using the established nomogram. The nomogram’s discriminating ability was assessed using the concordance index (C-index) and the receiver operating characteristic (ROC) curve. Calibration plots and decision curve analysis (DCA) were utilized to evaluate the nomogram’s predictive potential and clinical value.

### Survival analysis of patients with high-risk and low-risk score based on nomogram

Using the R software tool maxstat (Maximally selected rank statistics with various p-value approximations, version: 0.7–25), the optimal risk score cut-off value for the entire dataset was calculated. The minimum and maximum numbers of samples in each category were set at more than 25% and less than 75%, respectively. Based on available information, patients were separated into two groups based on their risk score: high risk and low risk. The Survfit function of the R software package was used to investigate the prognosis difference between the two groups, and the significance of the prognostic difference between the various groups of samples was tested using the log-rank test.

### Statistical analysis

SPSS v24.0 (SPSS Inc., USA) was used for all statistical analyses. The ideal cutoff values for age, blood platelet count and lymphocyte count, serum haemoglobin and albumin values, urinary specific gravity, leucocyte counts, bacterium, mucus filament and HALP score were determined using X-tile software v3.6.1 (Yale University) [13]. For continuous variables, the maximum, median, and lowest values were calculated; for categorical variables, frequencies and proportions were calculated. Using univariate and multivariate Cox regression analysis, the relevant hazard ratios (HRs) and 95% confidence intervals (CIs) were calculated. The “ggDCA” R package was used for DCA. To determine the area under the curve (AUC), ROC analysis was performed using the R software package pROC (version 1.17.0.1). We specifically collected the patients’ follow-up duration and risk score and did ROC analysis at 6, 12, 24, and 36 months using the ROC function of pROC. A *p* value of 0.05 was considered statistically significant for all analyses.

## Results

### Characteristics of the study cohort

The study population consisted of 91 patients with initially diagnosed NMIBC after TURBT. Table 1 summarizes all clinico-pathological features. The following cutoff values were noticed based on the findings obtained using X-tile software: 82 years for age, 35.26 for HALP, 134 g/L for hemoglobin, 37.5 g/L for albumin, 1.66 10^9^/L for lymphocyte count, 200 10^9^/L for platelet count, and 3.96 /uL for urinary leucocyte counts, 202 /uL for urinary bacterium, 178 /uL for urinary mucus filament. The median age of enrolled patients was 68 years old, the male-to-female ratio was approximately 4:1. The majority of patients, 67.03%, had tumors measuring less than 3 cm in diameter, while 43.96% had two or more tumors. Non-pedicled tumors were found in 37.63% of patients. Pathological examination revealed that 69.23% of the tumors were pT1 and 74.73% were high-grade diseases. Recurrence occurred in all patients in the entire study population, with a median follow-up period of 12 months.

**Table 1:** Representativeness of study participants

	Clinical characteristics	Number
Age	Median [min-max]	68 [43.00,92.00]
	≤ 82	79 (86.81%)
	> 82	12 (13.19%)
Gender	Female	18 (19.78%)
	male	73 (80.22%)
Urinary leucocyte counts (/uL)	Median [min-max]	10.56 [0–3979.9]
	≤ 3.96	21 (23.08%)
	> 3.96	70 (66.92%)
Urinary bacterium (/uL)	Median [min-max]	77.88 [0–1650]
	≤ 202	72 (79.12%)
	> 202	19 (20.88%)
Urinary mucus filament (/uL)	Median [min-max]	77.88 [0–5016]
	≤ 178	58 (63.74%)
	> 178	33 (36.26%)
Urinary nitrite	Negative	78 (85.71%)
	Positive	13 (14.29%)
Urinary protein	Negative	37 (34.07%)
	+	39 (42.86%)
	++	15 (16.48%)
T-stage	Ta	28 (30.77%)
	T1	63 (69.23%)
Tumor size (cm)	≤ 3	61 (67.03%)
	> 3	30 (32.97%)
Tumor grade	Low	23 (25.27%)
	High	68 (74.73%)
Tumor numbers	Single	51 (56.04%)
	Multiple	40 (43.96%)
Non-pedicled tumor	No	57 (62.64%)
	Yes	34 (37.36%)
Hemoglobin (g/L)	Median [min-max]	141 [102–181]
	≤ 134	27 (29.67%)
	> 134	64 (70.33%)
Albumin (g/L)	Median [min-max]	42.5 [33.5–51.7]
	≤ 37.5	10 (10.99%)
	> 37.5	81 (89.01%)
Lymphocyte (10^9^/L)	Median [min-max]	1.93 [0.69–3.87]
	≤ 1.66	27 (29.67%)
	> 1.66	64 (70.33%)
Platelet (10^9^/L)	Median [min-max]	210 [94–417]
	≤ 200	32 (35.16%)
	> 200	59 (64.84%)
HALP	Median [min-max]	56.77 [19.42–145.63]
	≤ 35.26	14 (15.38%)
	> 35.26	77 (84.62%)
Time to recurrence (months)	Median [min-max]	12 [3.00–66.00]

### Construction and internal validation of the nomogram

**Fig. 1:**
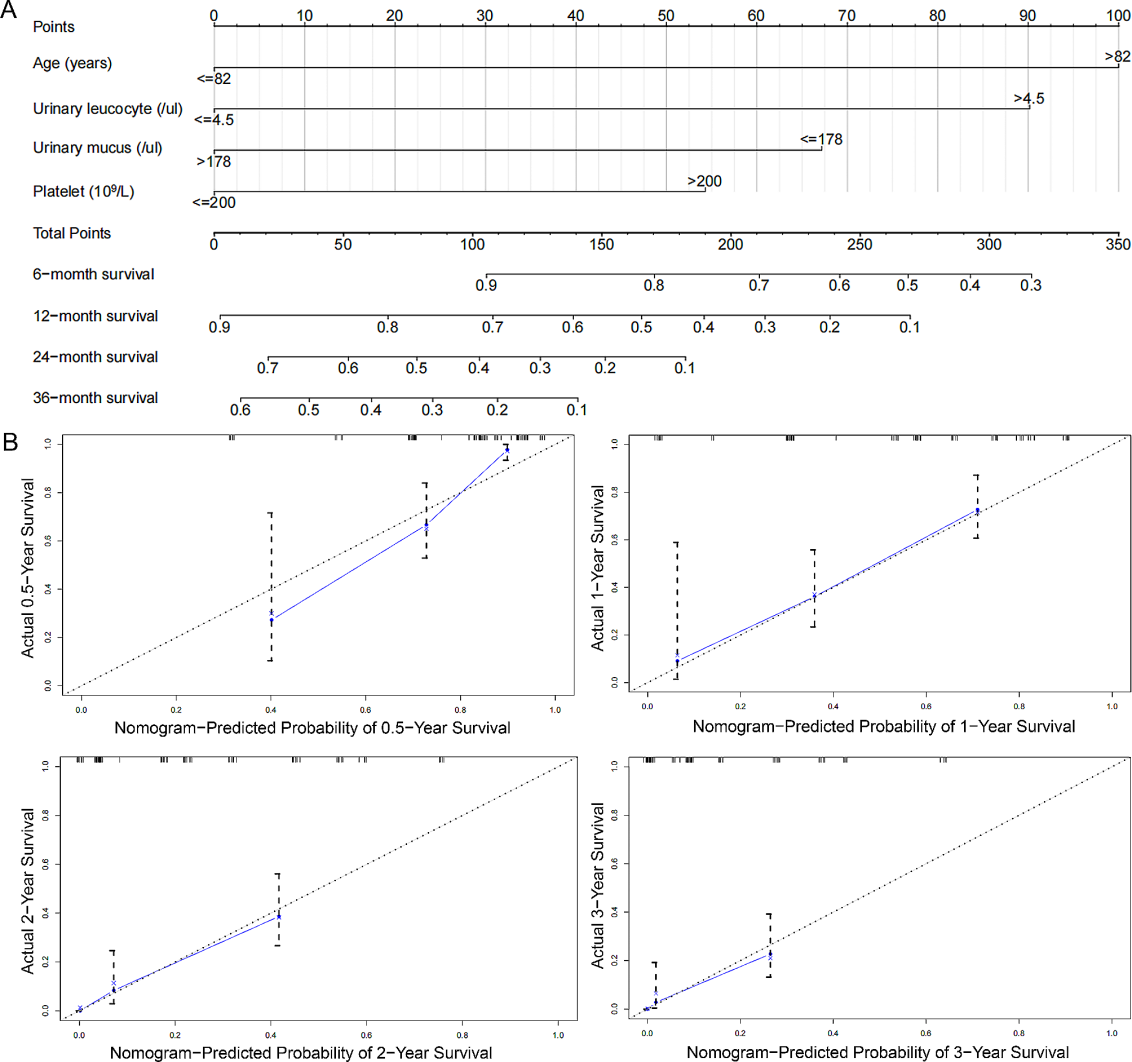
Establishment of a combined nomogram for customized prediction in recurrence of primary NMIBC. (A) Nomogram based on age, blood platelet count, urine leukocyte count and mucus filament was constructed to predict the 0.5-, 1-, 2- and 3-year recurrence free survival. (B) Predictive accuracy of the nomogram was assessed by the calibration plots

**Fig. 2:**
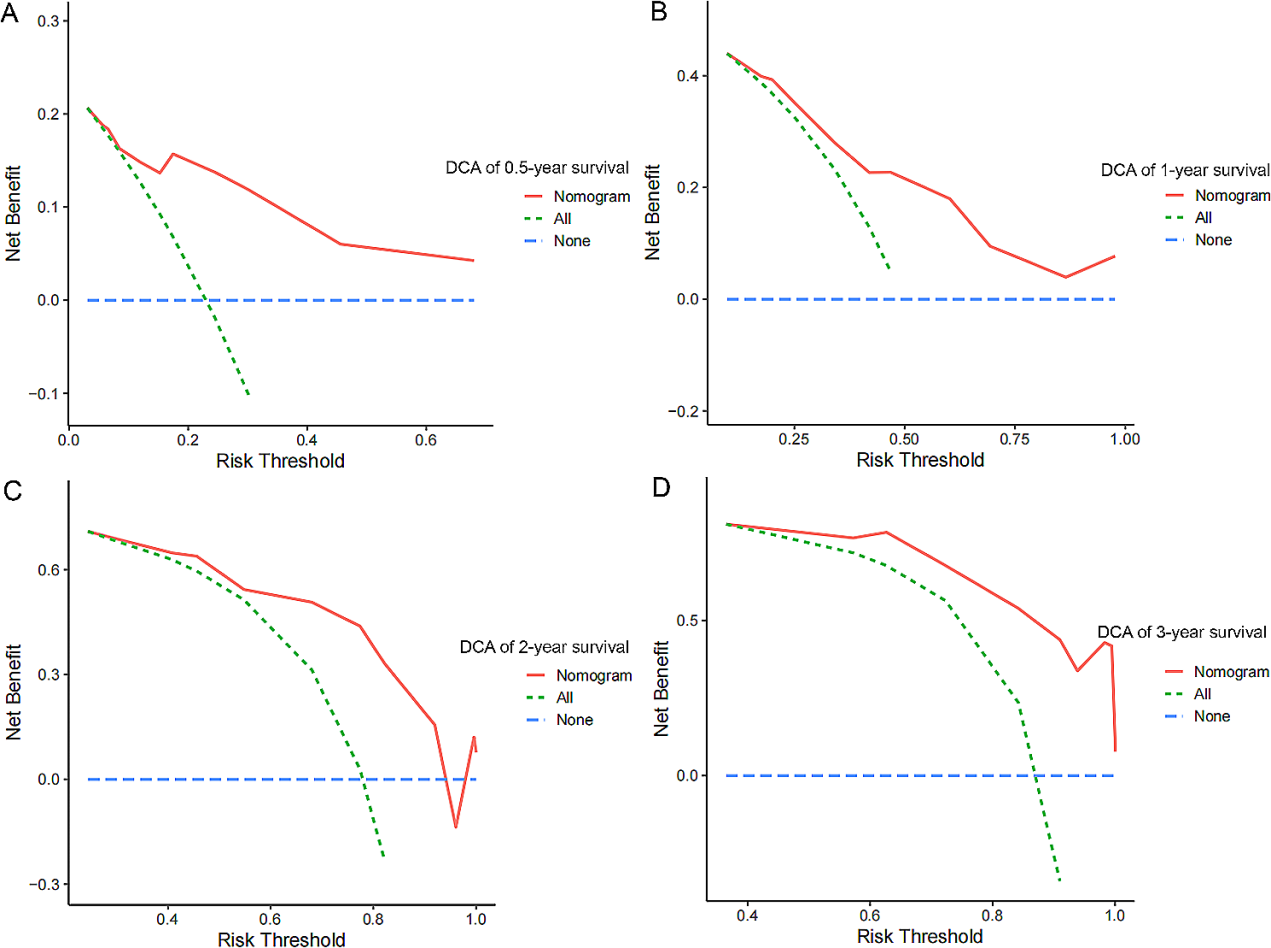
Predictive capacity of the nomogram was assessed by DCA. Decision curve analysis for 0.5-year (A), 1-year (B), 2-year (C) and 3-year (D) recurrence free survival

**Fig. 3:**
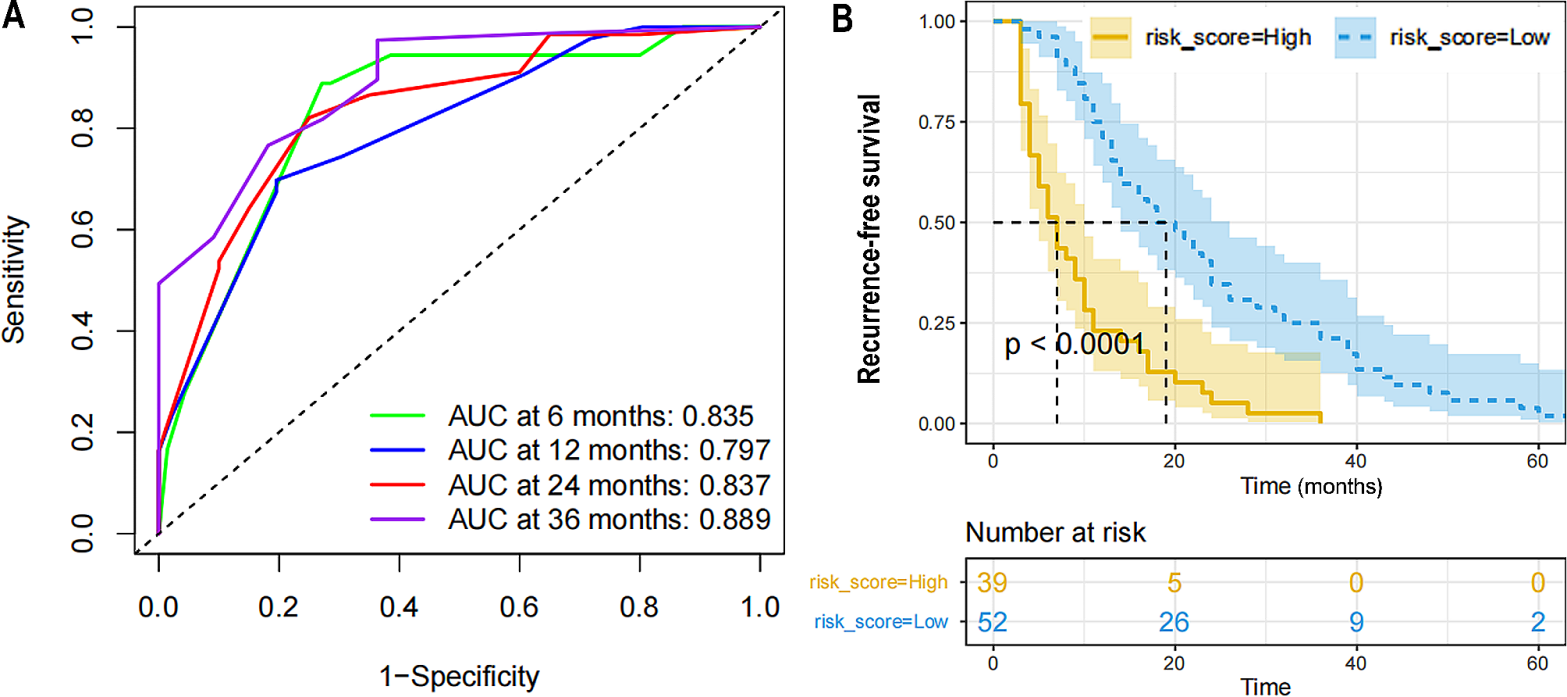
Validation of predictive value of the Nomogram. (A) The prognostic value of the nomogram was confirmed by the ROC analysis. (B) The recurrence free survival curves based on nomogram correlated risk score in the whole cohort

Table 2 summarizes the regression analysis results using univariate and multivariate Cox proportional hazard models. Age (HR: 2.33, *p* = 0.0330), urinary leucocyte counts (HR: 2.32, *p* < 0.0073), urinary mucus filament (HR: 0.42, *p* = 0.0006) and blood platelet count (HR: 1.70, *p* = 0.0337) were the independent risk factors for disease recurrence. A nomogram (Fig. 1A) was constructed using multivariate Cox regression analysis, and the C-index was 0.724, suggesting good consistency. The calibration plots (Fig. 1B) demonstrated that the nomogram could predict the NMIBC recurrence accurately. DCA curve revealed that the nomogram provided obvious net benefit at 6, 12, 24 and 36 month across a wide range of threshold probabilities, which represented patients correctly treated (Fig. 2). As shown in the ROC curve, the 6-month, 12-month, 24-month and 36-month AUC of the nomogram were 0.835, 0.797, 0.837 and 0.889, respectively (Fig. 3A).

**Table 2:** Univariate and multivariate analyses of factors associated with primary NMIBC recurrence

Variable		Univariate analysis		Multivariate analysis	
		HR (95%CI)	P value	HR (95%CI)	P value
Age	≤ 82 vs. > 82	3.94 (2.04–7.60)	*P* < 0.001***	2.33 (1.07–5.09)	0.0330*
Gender	Male vs. Female	1.26 (0.75–2.12)	0.385	-	
Urinary leucocyte counts (/uL)	≤ 3.96 vs. > 3.96	3.25 (1.84–5.76)	*P* < 0.001***	2.32 (1.26–4.32)	0.0073**
Urinary bacterium (/uL)	≤ 202 vs. > 202	1.88 (1.11–3.16)	0.0184*	1.53 (0.80–2.89)	0.1964
Urinary mucus filament (/uL)	≤ 178 vs. > 178	0.38 (0.24–0.61)	*P* < 0.001***	0.42 (0.25–0.69)	0.0006***
Urinary nitrite	Negative vs. Positive	1.03 (0.57–1.87)	0.92	-	
Urinary protein	Negative	-	-	-	
	+	0.98 (0.62–1.55)	0.9277	0.92 (0.54–1.55)	0.7497
	++	2.25 (1.20–4.22)	0.0112*	1.50 (0.73–3.06)	0.2607
T-stage	Ta vs. T1	1.18 (0.75–1.86)	0.465	-	-
Tumor size (cm)	≤ 3 vs. > 3	1.45 (0.93–2.26)	0.1	-	-
Tumor grade	Low vs. High	1.22 (0.76–1.98)	0.402	-	-
Tumor numbers	Single vs. Multiple	1.37 (0.89–2.07)	0.145	-	-
Non-pedicled tumor	No vs. Yes	1.33 (0.86–2.06)	0.199	-	-
Hemoglobin (g/L)	≤ 134 vs. > 134	0.73 (0.47–1.16)	0.192	-	-
Albumin (g/L)	≤ 37.5 vs. > 37.5	0.36 (0.18–0.72)	0.0041**	0.76 (0.35–1.71)	0.5177
Lymphocyte (10^9^/L)	≤ 1.66 vs. > 1.66	0.69 (0.43–1.10)	0.123	-	-
Platelet (10^9^/L)	≤ 200 vs. > 200	1.76 (1.11–2.78)	0.0154*	1.70 (1.04–2.78)	0.0337*
HALP	≤ 35.26 vs. > 35.26	0.44 (0.24–0.78)	0.0056**	0.72 (0.37–1.39)	0.3358

*P < 0.05, **P < 0.01, ***P < 0.001

Through nomogram modeling, all patients in this study were turned into a single risk score. Furthermore, the best risk score cutoff value was calculated as 2.84. The minimum sample size was chosen to be larger than 25%, while the maximum sample size was set to be less than 75%. Patients were divided into high and low risk groups based on this information, with 42.9% (*n* = 39) in the high risk group and 57.1% (*n* = 52) in the low risk group. There was a statistically significant difference in prognosis between the two groups (Fig. 3B).

## Discussion

In this study, we evaluated the ability of the routine blood and urine parameters in predicating the recurrence of primary NMIBC. Specifically, higher age, blood platelet count, urinary leucocyte counts and lower urinary mucus filament showed significant associations with rapid NMIBC recurrence. Furthermore, we established a nomogram model, which comprised the parameters above and performed well in the customized prediction of NMIBC recurrence at 6th, 12th, 24th and 36th month, hypothesized that its predictive value would assist doctors in identifying high-risk NMIBC patients and providing suitable follow-up alternatives.

The current gold standards for BC follow-up are urine cytology and cystoscopy with biopsy. However, cystoscopy is intrusive, expensive, has an accuracy rate of 85–90%, and has a risk of urinary tract infection, hematuria, and inadequate adherence to care guidelines. Additionally, it can be challenging to schedule repeated postoperative cystoscopic follow-ups for patients with NMIBC.

An extensive effort has been made in recent years to find biomarkers in blood and urine that can be used to diagnose BC and predict how well patients will respond to treatment. Lower urine pH and greater levels of urine protein, urine glucose, and pee occult blood have been linked to an increased risk of BC, according to research by Zeng et al. [9]. When compared to healthy controls, patients with bladder cancer have greater levels of urine- and plasma-soluble proteins such as VEGF, endostatin, stress proteins, and cytokines, which aid in the detection and staging of the disease [10]. After BCG injection, leukocyte cell presence in urine appears to be a replacement urine biomarker of immune system activity [11]. Additionally, a worse response to neoadjuvant chemotherapy has been associated to an increased neutrophil to lymphocyte ratio in MIBC patients’ blood [12]. However, few studies have looked into the use of non-invasive markers in blood or urine to predict the prognosis of NMIBC patients. Elsawy et al. reported that urinary IL-10 and serum tumor necrosis factor-α (TNF-α) can significantly predict the initial complete response after BCG treatment in high-risk NMIBC [14]. Li et al. observed that the presence of ferrous protoporphyrin(+)/reactive oxygen species(+) in urine is associated with an increased risk of recurrence in newly diagnosed NMIBC patients [15].

The HALP score has been identified as a significant predictive factor in patients with various malignancies and is thought to be an easily computed index of systemic inflammation and nutritional status [8, 9]. It was clear from these observations that platelets might be an unfavorable risk factor, but hemoglobin, albumin, and lymphocytes may be favorable risk factors. For patients with MIBC undergoing radical cystectomy, HALP score was indicated as an independent predictive predictor [16, 17]. However, it is unknown whether the HALP can predict recurrence of NMIBC patients. In addition, platelets have been shown to interact with cancer cells and aid their survival and metastasis through different mechanisms. By accumulating platelets, tumor cells can escape from the human immune system. And platelets could protect tumors from TNF-α mediated cytotoxicity and the high shear forces which could potentially damage them in flowing blood [18, 19]. According to our findings, though HALP score has no significant correlation with the prognosis of NMIBC, blood platelet count was the independent predictive factors for disease recurrence.

Numerous epidemiological investigations have looked into the link between Urinary tract infections (UTIs) and the risk of BC. The majority of these investigations supported the idea that recurring UTIs increase the risk of BC in the future [20, 21]. Jhamb et al. [22] and Jiang et al. [23] indicate, in contrast to these results, a decreased risk of BC with an increase in kidney and bladder infections. Uncertainty still exists regarding the prognosis of NMIBC and UTIs. In this study, we measured whether infection associated urine parameters were related to the prognosis of BC, including urinary leucocyte counts, nitrite, protein, bacterium and mucus filament. The results demonstrated that lower urinary mucus filament were related to rapid NMIBC recurrence. Interestingly, those with higher urine leucocyte levels had a higher recurrence risk. Similarly, Wong et al. found that a greater lymphocyte count in MIBC patients’ urine was substantially linked with illness recurrence [24].

Due to its user-friendly interface and numerical probability, the nomogram has been frequently employed to generate prognostic information for the specific patient [25]. With the aid of multivariate Cox regression analysis, an unique nomogram was created in this work by combining age, blood platelet count, urinary leucocyte counts and lower urinary mucus filament. The combined nomogram worked well as an all-encompassing scoring system for predicting the recurrence of NMIBC, which was supported by the ROC curve, calibration curve and C-index. DCA curve also demonstrated positive net benefits in guiding clinical decisions. Anymore, after dividing the patients into groups with low- and high-risk score according to the nomogram, the group with a high-risk score recurred rapidly and had a bad prognosis.

Although newer methods like fluid biopsy and genetic testing seem to increase the accuracy of BC prognosis predictions, they have drawbacks including a hefty price tag and labor-intensive analysis [26–28]. The majority of them are also still through clinical studies. Age, blood platelet count, urine leucocyte counts, and lower urinary mucus filament are routinely evaluated and reported for hospitalized patients in clinical practice, therefore there was no added cost or patient annoyance. This study is limited by a small sample size, which must be increased for future validation.

## Conclusions

Our study indicated significant association between routine blood and urine parameters and primary NMIBC recurrence within 6 months, 12 months, 24 months and 36 months. Specifically, higher age, blood platelet count, urinary leucocyte counts and lower urinary mucus filament showed significant associations with rapid NMIBC recurrence. Further, a novel combination nomogram for personalised risk prediction of NMIBC patients was developed and validated. Although the prognostic ability of this nomogram has been preliminary confirmed, it still requires further validation through clinical research, which will be our future effort.

## Data Availability

The data analyzed during the current study are available from the corresponding author on reasonable request.
